# Responses of bryosphere fauna to drought across a boreal forest chronosequence

**DOI:** 10.1007/s00442-022-05255-z

**Published:** 2022-09-08

**Authors:** Roger Grau-Andrés, Sylvia Thieffry, Shanyi Tian, David A. Wardle, Paul Kardol

**Affiliations:** 1 Department of Forest Ecology and Management, Swedish University of Agricultural Sciences (SLU), Umeå, Sweden; 2 Soil Ecology Lab, College of Resources and Environmental Sciences, Nanjing Agricultural University, Nanjing, 210095 China; 3 Asian School of the Environment, Nanyang Technological University, Singapore, Singapore

**Keywords:** Climate change, Mites, Moss, Nematodes, Precipitation

## Abstract

Projected changes in precipitation regimes can greatly impact soil biota, which in turn alters key ecosystem functions. In moss-dominated ecosystems, the bryosphere (i.e., the ground moss layer including live and senesced moss) plays a key role in carbon and nutrient cycling, and it hosts high abundances of microfauna (i.e., nematodes and tardigrades) and mesofauna (i.e., mites and springtails). However, we know very little about how bryosphere fauna responds to precipitation, and whether this response changes across environmental gradients. Here, we used a mesocosm experiment to study the effect of volume and frequency of precipitation on the abundance and community composition of functional groups of bryosphere fauna. *Hylocomium splendens* bryospheres were sampled from a long-term post-fire boreal forest chronosequence in northern Sweden which varies greatly in environmental conditions. We found that reduced precipitation promoted the abundance of total microfauna and of total mesofauna, but impaired predaceous/omnivorous nematodes, and springtails. Generally, bryosphere fauna responded more strongly to precipitation volume than to precipitation frequency. For some faunal functional groups, the effects of precipitation frequency were stronger at reduced precipitation volumes. Context-dependency effects were found for microfauna only: microfauna was more sensitive to precipitation in late-successional forests (i.e., those with lower productivity and soil nutrient availability) than in earlier-successional forests. Our results also suggest that drought-induced changes in trophic interactions and food resources in the bryosphere may increase faunal abundance. Consequently, drier bryospheres that may result from climate change could promote carbon and nutrient turnover from fauna activity, especially in older, less productive forests.

**Supplementary Information:**

The online version contains supplementary material available at 10.1007/s00442-022-05255-z.

## Introduction

Understanding the response of soil fauna to environmental change is key to predicting how global change affects ecosystem processes (Wardle et al. 2004; Wagg et al. 2014; Delgado-Baquerizo et al. 2020). This is because soil fauna plays a major role in carbon and nutrient cycling (Ingham et al. 1985; Filser 2002; de Vries et al. 2013; van den Hoogen et al. 2019), and because this role is often responsive to global change (Kardol et al. 2010; Bardgett and van der Putten 2014; Yin et al. 2019a). However, relatively little is known about the fauna inhabiting the bryosphere (i.e., the ground moss layer including live and senesced moss, and their associated biota; Lindo and Gonzalez 2010) and how they respond to global change.

The bryosphere is a key driver of net primary production, nutrient cycling, and decomposition in many ecosystems worldwide, and particularly in high-latitude ecosystems such as tundra, wetlands and boreal forests where the ground is often largely covered by mosses (Turetsky et al. 2010; Lindo et al. 2013; Street et al. 2013; Jackson et al. 2013; Grau-Andrés et al. 2021a). Bryosphere fauna may influence these processes by impacting bryophyte productivity through herbivory (Schill et al. 2011), stimulation of microbial decomposition through litter breakdown and communition (Seastedt 1984), and top-down control of nitrogen-fixing cyanobacteria (Kardol et al. 2016) and possibly decomposer biota (Sackett et al. 2010; Heidemann et al. 2014). Further, faunal abundance in soils (and in the associated litter and moss layers; Berg et al. 1998; Jonsson et al. 2015) of high-latitude ecosystems is particularly high due to the large stocks of soil organic matter in high-latitude regions (van den Hoogen et al. 2019), where climate change is also projected to be strongest (Collins et al. 2013). Therefore, bryosphere fauna may be an important driver of ecosystem responses to climate change in many high-latitude ecosystems. However, we know little about how the bryosphere fauna responds to climatic change factors such as altered precipitation regimes.

As climate change proceeds, droughts are projected to become more intense and frequent due to more variable precipitation regimes, and because of increased ground evaporation with warming (Collins et al. 2013; Berg et al. 2017). In soils, drought generally reduces the abundance and diversity of micro- and mesofauna (Lindberg et al. 2002; Kardol et al. 2010, 2011; Makkonen et al. 2011; Blankinship et al. 2011), although some studies have also recorded enhanced or neutral responses (Holmstrup et al. 2013, 2017; Sylvain et al. 2014; Turnbull and Lindo 2015). Reduced abundance and diversity of nematodes, mites and springtails have often been associated with a subsequent impairment of soil nitrogen mineralization and carbon turnover (Ingham et al. 1985; Filser 2002; Wall et al. 2008; Ferris 2010; de Vries et al. 2013; Bardgett and van der Putten 2014; Delgado-Baquerizo et al. 2020). While the effects of drought on soil fauna are widely studied, very few studies have investigated the effect of drought on bryosphere fauna (Lindo et al. 2012; Barreto and Lindo 2018). This is despite the fact that changes in moisture in the bryosphere under drought can be much more pronounced than in soil (Grau-Andrés et al. 2021b). Responses to drought may, however, vary between groups of fauna due to their differences in drought tolerance. For example, nematodes, which depend heavily on moisture as they live in water films on bryophyte tissues (Coleman et al. 2018a), may be more susceptible to drought than larger, more mobile micro-arthropods (Kardol et al. 2010, 2011). As a result, drought may impact both the abundance and community composition of bryosphere fauna.

Bryospheres are ubiquitous in boreal forests, which cover 27% of the global forest area and exert substantial influence on the global carbon cycle (Hansen et al. 2010; Gauthier et al. 2015), but studies on bryosphere fauna in boreal forests are scarce. However, Lindo et al. (2012) found large effects of experimental drying on springtail and mite abundance and composition in the bryosphere of *Pleurozium schreberi*, which is a widespread feather moss species in boreal forests. Importantly, environmental conditions in boreal forests can vary strongly along successional gradients (Gundale et al. 2009). Compared to early-succession boreal forests, late-succession forests are dominated by resource-conservative vascular plants (i.e., those that are less productive and produce lower quality litter) and soil fungi (which produces more recalcitrant necromass), and have less fertile soils with lower mesofaunal abundance (Wardle et al. 2003, 2012; Clemmensen et al. 2013; Bokhorst et al. 2014). Some studies have shown that precipitation effects on fauna can depend on environmental context (de Vries et al. 2012; Yin et al. 2019b). For example, stronger drought effects on micro- and mesofauna have been observed in ecosystems supporting more bacterial-based relative to more fungal-based soil food webs (de Vries et al. 2012). As such, variation in environmental conditions in the boreal forest floor is likely to shape bryosphere faunal communities (Bokhorst et al. 2014) and their response to drought.

Here, we carried out a greenhouse mesocosm experiment to assess the effects of the precipitation regime on the abundance and community composition of microfauna (nematodes and tardigrades) and mesofauna (mites and springtails) in boreal forest bryospheres. To test the role of environmental context in driving these effects, we used bryospheres and upper humus layers collected from 30 well-characterised forested lake islands in northern Sweden which collectively represent a chronosequence across which soil fertility, litter quality inputs into the bryosphere, and moss nutrient content all show large changes (Wardle et al. 2012). We subjected these bryospheres to a factorial combination of two levels (ambient and reduced) of water addition volume and frequency, to create variation in both moisture levels and wetting–drying cycles. We hypothesised that: (1) Drier conditions reduce bryosphere faunal abundance and diversity, and shift the community towards a greater abundance of mesofauna relative to microfauna, and of non-predaceous fauna relative to predaceous fauna. This is because drought may have stronger negative effects on microfauna, which live in water films, than on more mobile mesofauna (Sylvain et al. 2014), and on larger-bodied fauna at high trophic levels than on smaller-bodied fauna at low trophic levels (Franco et al. 2019); (2) Effects of reduced precipitation frequency are greater at reduced precipitation volumes because water stored in the lower bryosphere layers and humus keeps the bryosphere moister at ambient precipitation volumes (Grau-Andrés et al. 2021b); (3) Drought effects on bryosphere fauna are greater in early- compared to late-succession boreal forests. This is based on early-succession boreal forests having a soil food web that is more bacterial-based, and which would therefore be more sensitive to moisture variation, than would food webs that are more fungal-based (de Vries et al. 2012). By testing these hypotheses, we aimed to improve our understanding of bryosphere fauna responses to altered precipitation regimes across contrasting environmental conditions. This understanding is key to predicting climate change effects on ecosystem functions in contrasting moss-dominated ecosystems.

## Methods

### Sampling site

The bryosphere samples were collected from 30 forested lake islands in northern Sweden (lakes Hornavan and Uddjaure, N 65° 57ʹ to 66° 10ʹ, E 17° 43ʹ to 17° 52ʹ). Monthly average air temperature ranges between + 13 °C in July and − 14 °C in January, and mean annual rainfall is 750 mm. All islands originated about 9000 years ago upon the retreat of land ice, and the only major extrinsic factor that varies between the islands is wildfire frequency resulting from lightning strikes. Compared to larger islands, smaller islands are struck by lightning less often and are thus subjected to less frequent stand-replacing fires (Wardle et al. 1997). As such, islands range from those that last burned 60 years ago to those that last burned over 5000 years ago, resulting in a 5000-year time since fire chronosequence (Wardle et al. 2012). As time since fire increases with decreasing island size, there is a shift from resource-acquisitive to resource-conservative vascular plant and microbial communities, and a decline in vascular plant productivity, soil fertility, and rates of decomposition and nutrient fluxes (Wardle et al. 2003; Clemmensen et al. 2015; Kumordzi et al. 2015). Compared to larger islands, smaller islands have more resource-conservative plant communities that are less productive and produce a more recalcitrant litter and have more resource-conservative microbial communities (i.e., more fungal-based) that produce more recalcitrant necromass (Clemmensen et al. 2013; Lagerström et al. 2013). The proportion of the ground surface covered by feather mosses, which is almost entirely dominated by *Hylocomium splendens* and *Pleurozium schreberi*, is on average 41.2% and is constant throughout the chronosequence (Jonsson et al. 2015), while moss biomass, nitrogen fixation by moss-associated cyanobacteria, and moss tissue nitrogen are highest on the smaller islands (Lagerström et al. 2007; Bansal et al. 2012). Consistent with previous work in this study system (Wardle et al. 2003, 2012; Kardol et al. 2018; Fanin et al. 2018; Grau‐Andrés et al. 2020) we categorised the 30 islands into 10 ‘large’ islands representing early-successional forests (> 1.0 ha, mean time since fire: 585 years), 10 ‘medium’ islands representing intermediate-successional stages (0.1–1.0 ha, mean time since fire: 2180 years), and 10 ‘small’ islands representing late-successional stages (< 0.1 ha, mean time since fire: 3250 years) (see Table S1 for more details).

### Bryosphere sampling and experimental design

We collected four samples of the bryosphere and the underlying humus layer from each of the 30 islands (*N* = 120) between 30 July and 16 August 2018, using a 10.3-cm diameter corer fitted with a serrated edge. We sampled monospecific moss layers dominated by *Hylocomium splendens*, which is the most abundant moss species across all the islands. The samples included the upper, living moss and the lower, senesced moss (mean height ± SD was 4.3 ± 1.1 cm), and the slightly to moderately decomposed organic matter forming the top 5.7 ± 1.1 cm of the humus layer (i.e., Oi and/or Oe organic soil horizons; Soil Survey Staff 2015). The upper humus is likely to influence bryosphere fauna both directly through faunal exchange and indirectly through regulating moisture dynamics (Carleton and Dunham 2003; DeLucia et al. 2003; Lindo and Gonzalez 2010). Moss and humus thickness in the collected samples measured in the field did not vary between island size classes (*F*_*2,27*_ = 2.2, *P* = 0.13).

Upon collection, the samples were each placed in mesocosms made out of PVC cylinders (10.3 cm internal diameter, 10 cm height) fitted with a permeable bottom. Each mesocosm was covered with a permeable gardening cloth and kept moist at ambient temperature for 2–19 days, and then stored at 4 °C for 18 days. This storage temperature is recommended for the storage of soil fauna prior to experimentation (Coleman et al. 2018b) and is common in micro- and mesofaunal research (Lindberg et al. 2002; Jonsson et al. 2015; Kardol et al. 2016). On 6 September 2018, the mesocosms were transported to a greenhouse (Wallenberg Lab Greenhouse, Umeå Plant Science Centre, Umeå, Sweden) and subjected to one of four precipitation treatments (see below) until 20 December 2018 (104 days). The greenhouse photoperiod was 18 h/6 h, air temperature averaged 20.5 °C and relative humidity was 70%. Further details on the experimental set-up can be found in Grau-Andrés et al. (2021b).

### Water addition treatments

**Fig. 1:**
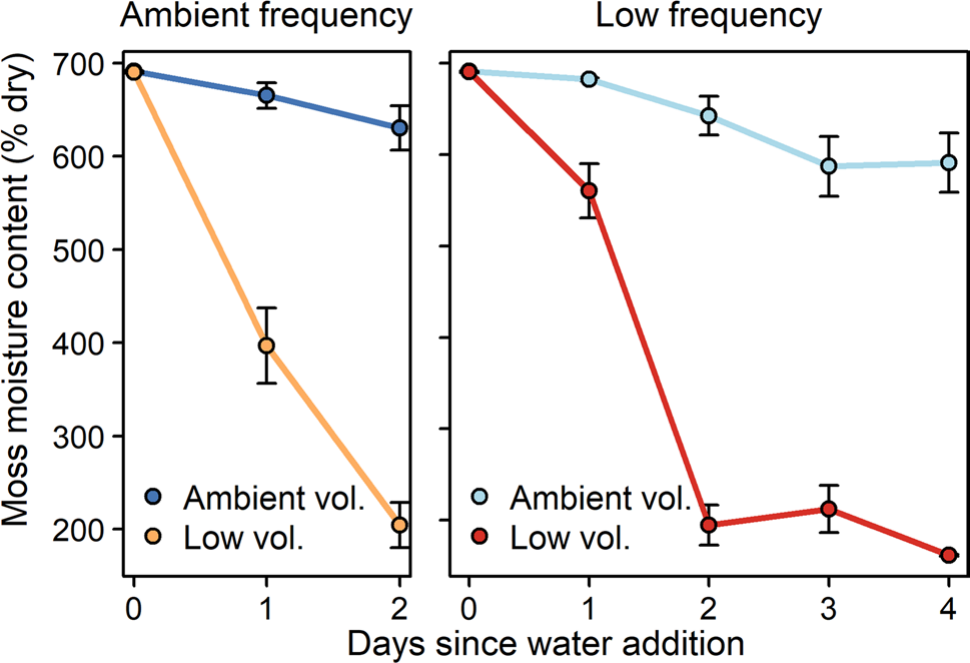
Moisture content (mean ± SE, on a % dry weight basis, *N* = 30) of the upper 2 cm moss layer in mesocosms subjected to ambient versus low volume of precipitation, in (left) ambient frequency treatments and (right) low frequency treatments. Moisture content was estimated using a combination of visual assessment of wet and dry moss cover and destructive sampling. Data from Grau-Andrés et al. (2021b)

Each of the four mesocosms per island was assigned to one of four precipitation treatments. The watering treatments were: 40 ml every 2 days, 10 ml every 2 days, 80 ml every 4 days, and 20 ml every 4 days. The treatments resulted from a factorial combination of two levels of water addition volume (either 20 ml or 5 ml per mesocosm per day on average) and two levels of water addition frequency (either every 2 days or every 4 days). The higher watering volume and frequency represented the approximate mean summer (June–September) precipitation regime at the sampling site (i.e., ambient conditions), while the lower volume and frequency treatments aimed to represent lower ground moisture due to projected longer droughts and increased ground evaporation in the region resulting from climate change (Grau-Andrés et al. 2021b). We used rain water collected in the vicinity of the greenhouse (DOC = 1.8 ± 0.1 mg C l^−1^, dissolved nitrogen = 0.15 ± 0.03 mg N l^−1^, *N* = 3), and applied it to the top of the mesocosms using a spray bottle. The mesocosms were loosely covered with a gardening mesh to lower evaporation rates and thus simulate the insulating effect of understory dwarf shrub and tree canopy on evaporation from the bryosphere (Heijmans et al. 2004) and to reduce the loss of mesofauna. As we showed in Grau-Andrés et al. (2021b), the upper moss remained wet throughout the watering cycle (irrespective of precipitation frequency) at ambient levels of precipitation volume, while in low-volume precipitation treatments higher rates of moss drying led to a generally drier moss layer (Fig. 1).

### Extraction of microfauna and mesofauna

At the end of the experiment, we separated the moss layer (including photosynthetic green tissue and non-photosynthetic ‘brown’ moss) from the underlying humus layer for each mesocosm and stored the moss in air-tight plastic bags at 4 °C for 12–18 days. We used modified Baermann funnels to extract microfauna (i.e., nematodes and tardigrades) from the moss layer as described by Jonsson et al. (2015). To do this, we placed moss sub-samples (consisting of about a quarter of each moss sample) onto separate funnels (*N* = 120) and submerged the moss with tap water. Every 24 h for 5 days we extracted about 10 ml of solution from each funnel, and stored it at 4 °C. After each extraction, we shook the moss and added water to ensure that all the moss was submerged. After the last extraction, the stored solution was left to settle at 4 °C for 24 h and then all but the bottom 2 ml were removed using a pipette. We then preserved the microfauna by adding 5 ml of 4% formaldehyde at 90 °C followed by 5 ml of cold formaldehyde. To extract mesofauna (i.e., mites and springtails), we placed a second set of sub-samples (each about half of the original moss samples) in Tullgren extractors (*N* = 120) for 5 days as described by Kardol et al. (2016). The extracted mesofauna was collected in 70 % ethanol solution. Finally, each moss sub-sample used for extracting micro- and mesofauna was oven-dried at 60 °C for 48 h and weighed.

### Characterisation of microfauna and mesofauna

For each sample, we counted all nematodes and all tardigrades in the entire extracted solution (or a known fraction of it for highly concentrated samples) using a grid-patterned petri dish mounted on a compound microscope. Additionally, we identified the first 150 nematodes that we found in each sample to family level (except the super-family Dorylaimoidea) following Bongers (1988), and used this information to estimate the total counts of each nematode taxa in the sample. The taxa were assigned to one of four trophic groups (i.e., plantivorous, fungivorous, bacterivorous, and predaceous/omnivorous) following Yeates et al. (1993) (Table S2). The super-family Dorylaimoidea (*sensu* Jairajpuri and Ahmad 1992) comprised the dorylaimid families Dorylaimidae, Qudsianematidae, Thornenematidae, and Aporcelaimidae, which are overall considered predaceous/omnivorous (Yeates et al. 1993). These four nematode groups, plus tardigrades (which feed on algae, moss tissues, micrometazoans, and organic matter rich in bacteria, and are considered to be plantivorous/omnivorous; Coleman et al. 2018a), resulted in five microfaunal functional groups (Jonsson et al. 2015; Kardol et al. 2016). To calculate mesofauna abundance, we counted the total number of mites (Acari) and springtails (Collembola) in each sample using a dissecting microscope. All mites were assigned to one of five functional groups based on taxonomy and feeding preference, i.e., Oribatida combined with Astigmata (fungivorous), Mesostigmata (predaceous), Prostigmata belonging to the family Tydeidae (fungivorous), and all other Prostigmata (predaceous), following Krantz and Walter (2009) and Walter and Proctor (2013). Juvenile mites that could not be assigned to a functional group formed a separate group. All springtails were considered fungivorous, as the predaceous subfamily Frieseinae (family Neanuridae) was absent in our samples (Holtkamp et al. 2008). We derived micro- and mesofauna abundance on a moss-weight mass basis by dividing the number of counts for each functional group by the dry weight of the moss sub-sample used for the faunal extractions.

The alpha diversity and the Shannon diversity index were calculated for each sample using the functions ‘specnumber’ and ‘diversity’, respectively, in the package *vegan* (Oksanen et al. 2020), separately for nematode taxa, microfaunal functional groups, and mesofauna functional groups. Since our functional groups are based on functional traits (i.e., morphological, behavioural), their diversity could indicate faunal functional diversity (Kamath et al. 2022). Additionally, we computed the ‘Maturity Index’ of the nematode community for each sample by calculating the weighted average of the colonizer to persister scores of all nematode taxa (Bongers 1990; Bongers and Ferris 1999). Low Maturity Index values indicate that the nematode community is dominated by early colonizers of new resources, while high scores are associated with persisters in undisturbed habitats. Colonizer–persister scores for each nematode taxa are given in Table S2.

### Statistical analyses

We used R version 4.1.1 (R Core Team 2021) for all statistical analyses and plotting. To assess the effect of precipitation regime and context-dependency on micro- and mesofauna communities in the bryosphere, we first analysed the abundance (i.e., number of individuals per dry weight of moss) of each of the micro- and mesofauna functional groups separately. To do this we fitted, for each group, a linear mixed effects model (function ‘lme’ in package *nlme*; Pinheiro et al. 2021) which included precipitation volume, precipitation frequency, island size class, and their interactions, as fixed effects. The identity of each of the 30 islands was included as a random effect to account for the spatial non-independence among the four mesocosms from each island. We fitted the exact same model to the response variables alpha diversity, Shannon diversity index (separately for nematode taxa, microfaunal functional groups, and mesofaunal functional groups), and the Maturity Index. Additionally, to test whether total microfaunal and total mesofaunal abundances differed in their responses to precipitation regime, we used a linear mixed effects model with total faunal (i.e., mesofaunal or microfaunal) abundance as the response variable, and precipitation volume, precipitation frequency, island size class, faunal group (i.e., microfauna or mesofauna), and their interactions, as fixed effects. Mesocosm nested within island identity was included as a random effect to account for the non-independence among mesocosms from the same island and among abundance measurements from the same mesocosm. We log-transformed the response variables and used a constant variance function (‘varIdent’) to account for variance heterogeneity among treatments and/or island size classes when appropriate (Zuur et al. 2009). Pairwise comparisons between precipitation treatments and island size classes were computed using the package *emmeans* (Lenth 2021).

Permutational multivariate analysis of variance (PERMANOVA; Anderson 2001) was used to test differences in micro- and mesofauna community composition based on functional groups. To do this, we first calculated dissimilarity matrices for our 120 mesocosms based on their community composition using the Jaccard dissimilarity index (Faith et al. 1987), separately for micro- and mesofauna. We then tested the effect of volume and frequency of precipitation, island size class, and their interaction, on each dissimilarity matrix using the function ‘adonis2’ in *vegan*. To account for the non-independence of samples taken from the same island, we restricted permutations to within islands. Non-metric multidimensional scaling (NMDS; as implemented in the function ‘metaMDS’ in *vegan*) was used to visualise variation in micro- and mesofauna community composition in relation to precipitation treatments and island size classes. The abundance data was first standardised by row and column maximums (i.e., Wisconsin double standardisation) as recommended by the function ‘metaMDS’. We selected two-dimension solutions to facilitate the interpretation of the ordination diagrams. The function ‘ordiellipse’ in *vegan* was used to draw the standard deviations around each water addition treatment.

## Results

### Main effects of precipitation on bryosphere fauna (Hypothesis 1)

**Fig. 2:**
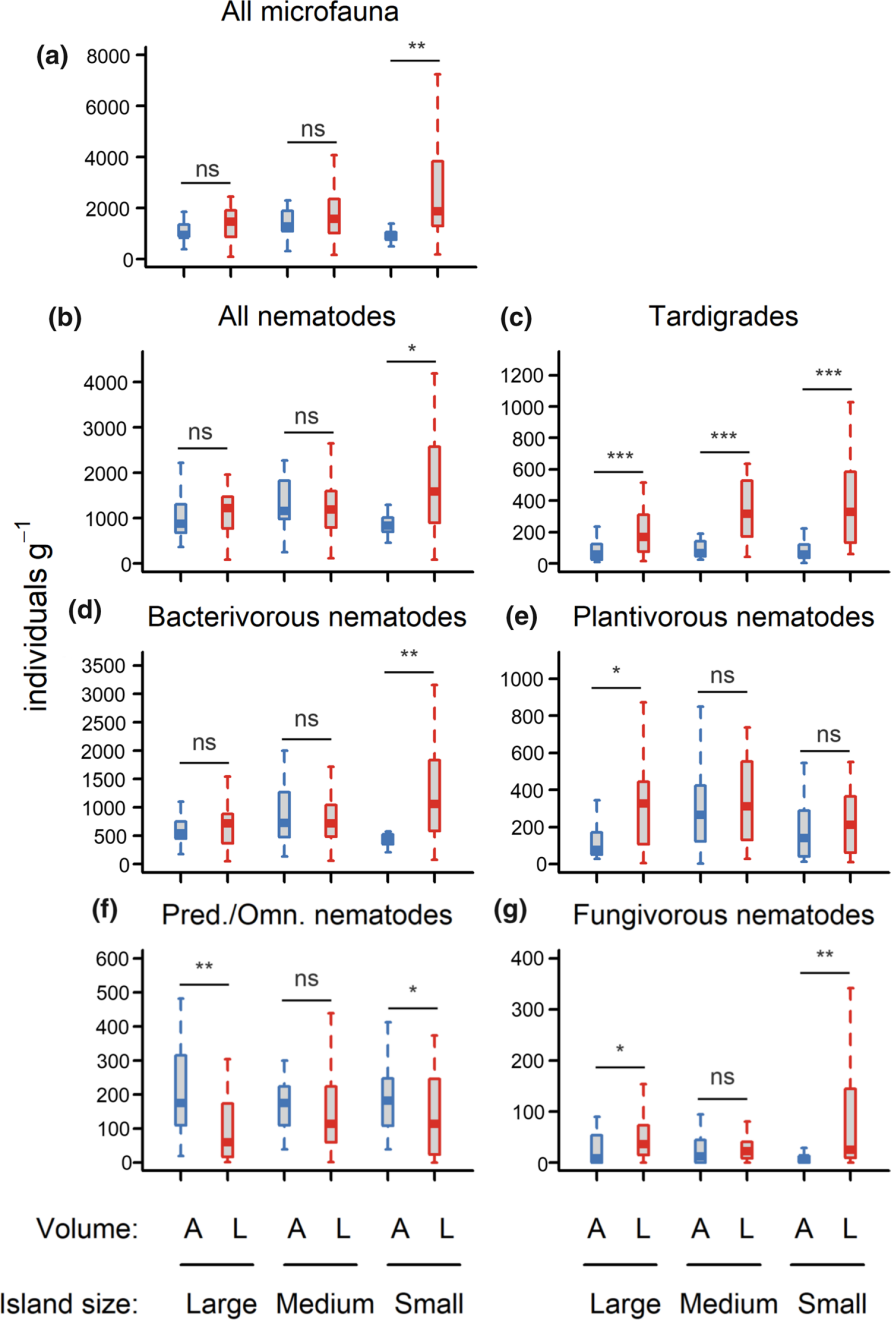
Microfaunal abundance (i.e., number of individuals per dry moss mass) per precipitation volume (Ambient (A) or Low (L)) and island size class (Large, Medium, Small), which were the most important factors driving microfauna abundance (Table S3). Box plots indicate the median (thicker line), the first and third quartiles (lower and upper box boundaries), and the most extreme observations that were up to 1.5 times the interquartile range (hinges). Data were aggregated across the frequency of water addition. For each volume × island size combination, *N* = 20. Within each panel and island size class, post hoc comparisons of volume treatments are indicated by ‘ns’ (*P* > 0.05), ‘*’ (*P* < 0.05), ‘**’ (*P* < 0.01), and ‘***’ (*P* < 0.001). Details of the model underpinning the statistical testing are provided in Table S3. *Omn./pred.* omnivorous/predaceous

**Fig. 3:**
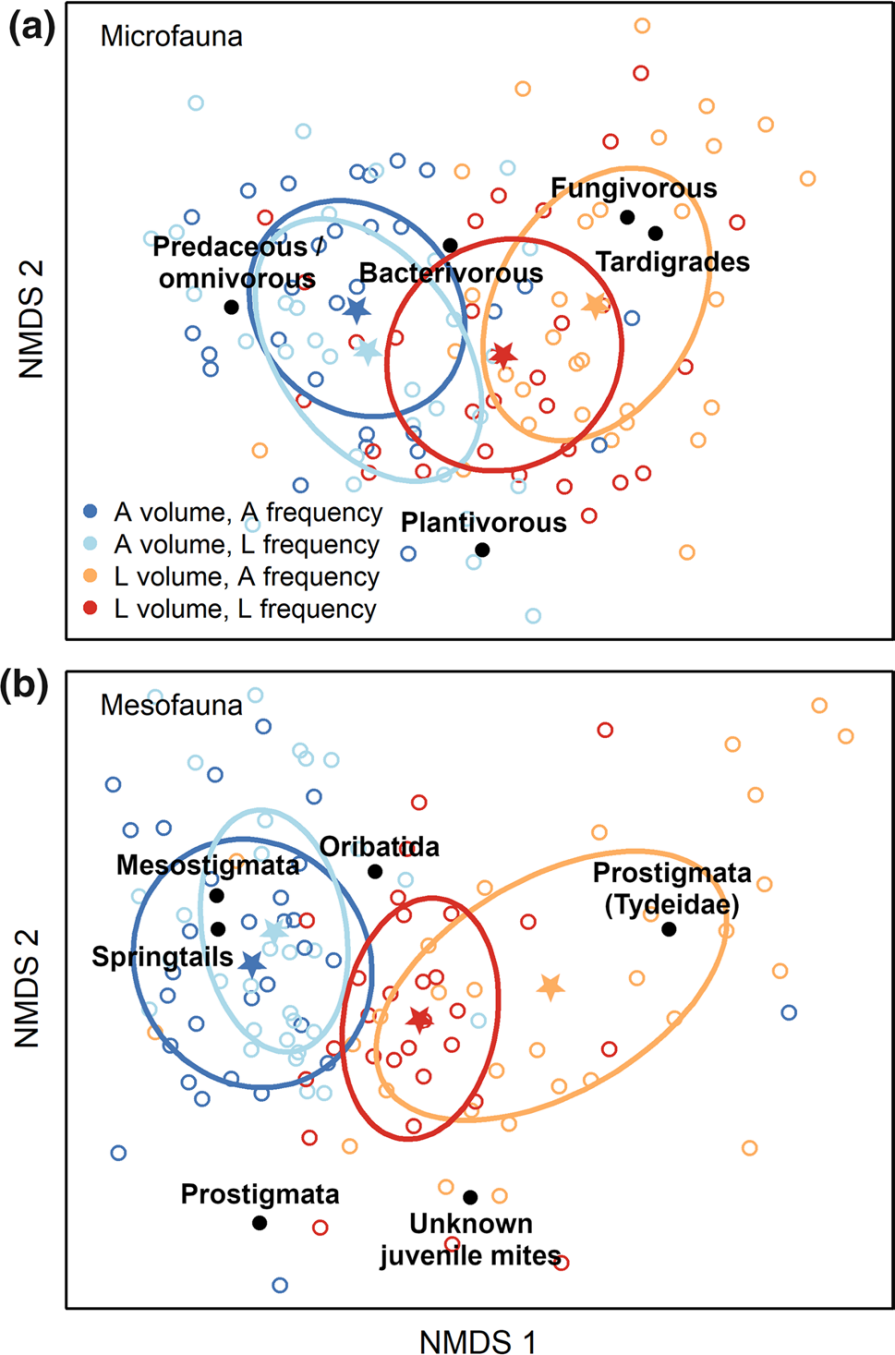
NMDS diagrams of the community composition of (**a**) microfaunal and (**b**) mesofaunal functional groups, for each combination of precipitation volume (‘A’, ambient and ‘L’, low) and frequency (‘A’, ambient and ‘L’, low). Microfauna includes nematodes (bacterivorous, plantivorous, predaceous/omnivorous, and fungivorous), and tardigrades. Mesofauna includes mites (Oribatida, Mesostigmata, Prostigmata belonging to the family Tydeidae, other Prostigmata, and unknown juveniles), and springtails. Open circles are samples, stars are centroids of water addition treatments, and ellipses are standard deviations of the treatment centroids. Stress values were 0.187 (microfauna), and 0.175 (mesofauna)

Bryosphere microfauna consisted of tardigrades (as a single group) and 15 different nematode taxa, of which Plectidae (bacterivorous), Tylenchidae (plantivorous) and Teratocephalidae (bacterivorous) were the most abundant (Table S2). Precipitation volume strongly impacted on total microfaunal abundance, and on abundance and alpha diversity of all microfaunal functional groups, while precipitation frequency had no main effects (Table S3, Table S4). Total microfaunal abundance was promoted by low precipitation volume through increasing the abundance of tardigrades and bacterivorous, plantivorous, and fungivorous nematodes (Fig. 2). Only the abundance of predaceous/omnivorous nematodes was impaired by low precipitation volume (Fig. 2f). Alpha diversity of microfaunal functional groups was promoted by low precipitation volume (Figure S1). PERMANOVA indicated that both precipitation volume and frequency affected microfaunal community composition based on functional groups, with volume having stronger effects (Table S5). Ordination analyses showed that microfauna community composition was structured along the main gradient of precipitation regime (Fig. 3a) which corresponds with bryosphere moisture content, from wettest (ambient volume and frequency) to lowest (low volume, ambient frequency) (Fig. 1). Wetter treatments were associated with predaceous/omnivorous nematodes, while drier conditions were more closely associated with greater dominance of tardigrades and fungivorous nematodes (Fig. 3a).

**Fig. 4:**
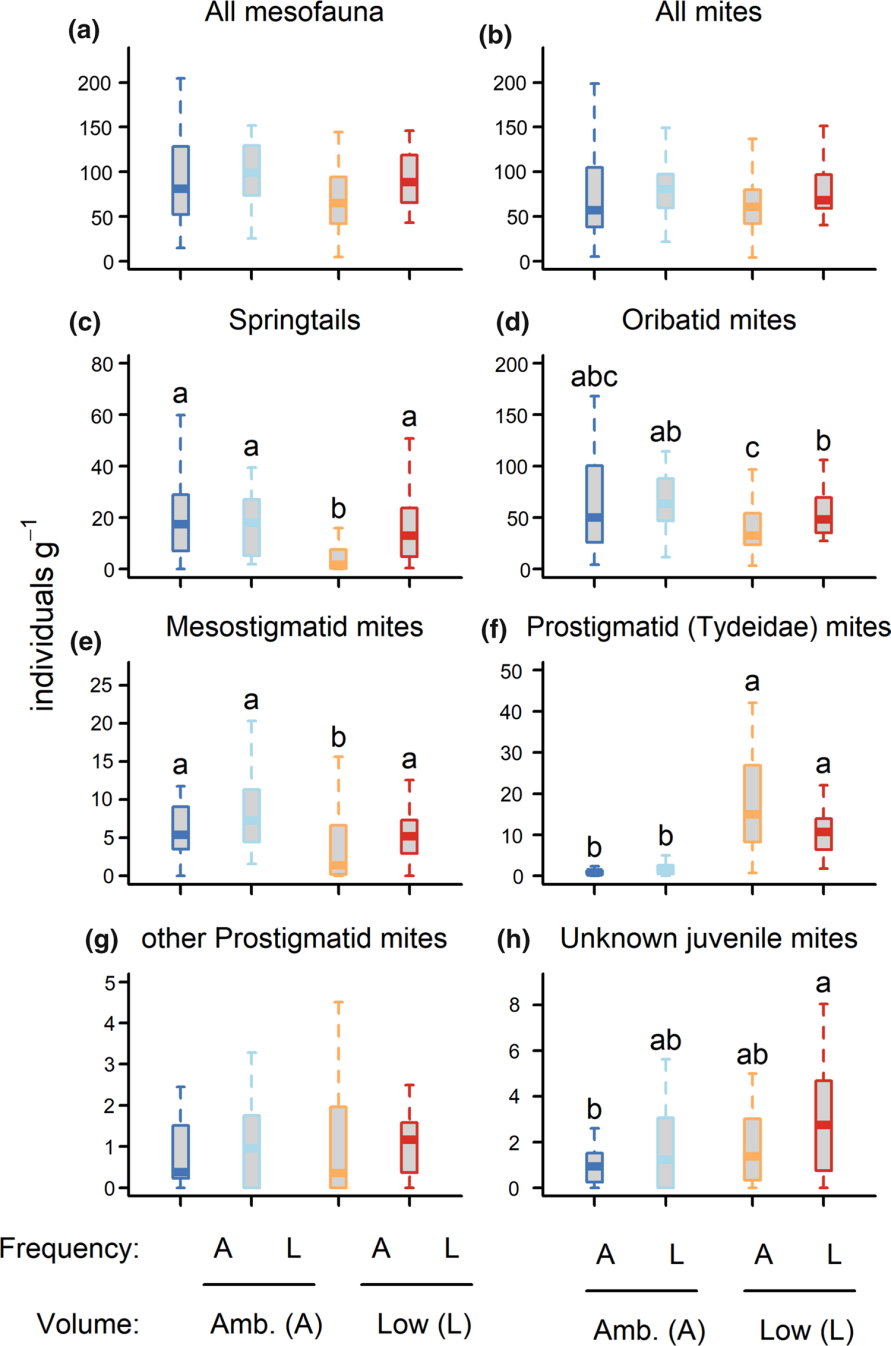
Mesofaunal abundance (i.e., number of individuals per dry moss mass) for each of two levels (Ambient (A) and Low (L)) of precipitation volume and frequency. Data were aggregated across island size class because island size had only minor effects on mesofauna (Table S5). Boxplots indicate the median (thicker line), the first and third quartiles (lower and upper box boundaries), and the most extreme observations that were up to 1.5 times the interquartile range (hinges). For each volume × frequency combination, *N* = 30. Within each panel, the same letters (or no letters) indicate that differences are not statistically significant (i.e., *P* > 0.05). Details of the model underpinning the statistical testing are provided in Table S5

The abundance of total mesofauna, total mites, and Oribatid mites all responded to precipitation frequency but not to volume (Table S6). Lower precipitation frequency promoted the abundance of those groups (Fig. 4). Conversely, the abundance of springtails, Prostigmatid (Tydeidae) mites and juvenile mites all responded to precipitation volume but not frequency (Table S6). Lower precipitation volume reduced the abundance of springtails but increased the abundance of Prostigmatid (Tydeidae) mites and juvenile mites (Fig. 4). Mesostigmatid mites were affected both by volume and frequency of precipitation (Table S6): lower precipitation volume decreased their abundance, while lower frequency increased it (Fig. 4). Shannon diversity of mesofauna increased with lower precipitation volume (Figure S1, Table S4). Similar to microfauna, PERMANOVA of mesofauna indicated that both volume and frequency of precipitation impacted community composition, with volume having stronger effects (Table S5). Ordination showed that bryospheres that received low precipitation volumes were associated with Prostigmatid (Tydeidae) mites and juvenile mites, while ambient precipitation volumes were associated with springtails, other Prostigmatid mites, and Oribatid mites.

Precipitation frequency affected the total abundance of microfauna and of mesofauna similarly (interaction of frequency × faunal group: *F*_*1,108*_ = 1.2, *P* = 0.279), but precipitation volume had a greater effect on microfauna than on mesofauna (interaction of volume × faunal group: *F*_*1,108*_ = 10.1, *P* = 0.002).

### Interactive effects of precipitation volume and frequency (Hypothesis 2)

**Fig. 5:**
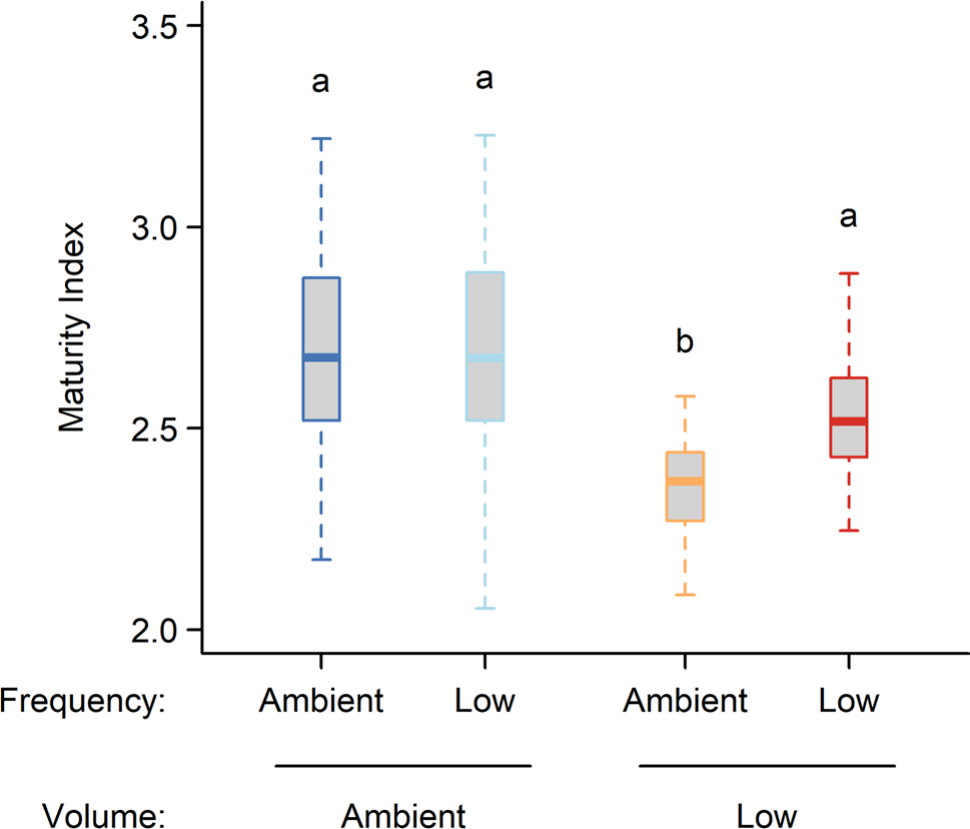
Maturity Index (based on the ‘c–p’ framework of Bongers 1990) of the nematode community for each of two levels (Ambient and Low) of precipitation volume and frequency. The index ranges from 1 (domination of community by early colonizers in disturbed habitats) to 5 (domination of community by persister taxa in undisturbed habitats). Data were aggregated across island size class because island size had only minor effects on the Maturity Index (Table S7). Boxplots indicate the median (thicker line), the first and third quartiles (lower and upper box boundaries), and the most extreme observations that were up to 1.5 times the interquartile range (hinges). For each volume × frequency combination, *N* = 30. Same letters indicate that differences are not statistically significant (i.e., *P* > 0.05). Details of the model underpinning the statistical testing are provided in Table S7

The abundance of total microfauna, and of all microfaunal functional groups except predaceous/omnivorous nematodes, did not respond to an interaction between volume and frequency of precipitation (Table S3). The interactive effects for predaceous/omnivorous nematodes occurred because lower precipitation frequency increased the abundance at low volumes, but had no effect at ambient volumes (data not presented). Similarly, the Maturity Index of the nematode community also responded to the interactive effect of volume and frequency whereby lower frequency increased the Maturity Index at low volumes, but had no effect at ambient volumes (Fig. 5, Table S7). Conversely, volume and frequency did not interact to drive microfaunal community composition, or diversity of nematode taxa or microfaunal functional groups (Table S4, Table S5).

Total mesofaunal abundance did not respond to the interactive effect of precipitation volume and frequency, but the abundance of springtails and of Prostigmatid (Tydeidae) mites did (Table S6). Springtail abundance was increased by lower precipitation frequency at low precipitation volumes, but frequency had no effect at ambient volumes (Fig. 4c). In contrast, the abundance of Prostigmatid (Tydeidae) mites was decreased by the lower frequency at low volumes but increased at high volumes (Fig. 4f). Mesofaunal alpha and Shannon diversity were not affected by the interactive effect of precipitation volume and frequency (Table S4). A marginally non-significant interaction (*P* = 0.053) between volume and frequency of precipitation was found by PERMANOVA (Table S4), suggesting that frequency may have affected mesofaunal community composition more strongly at low compared to ambient volumes (Fig. 3).

### Context-dependency of precipitation effects (Hypothesis 3)

**Fig. 6:**
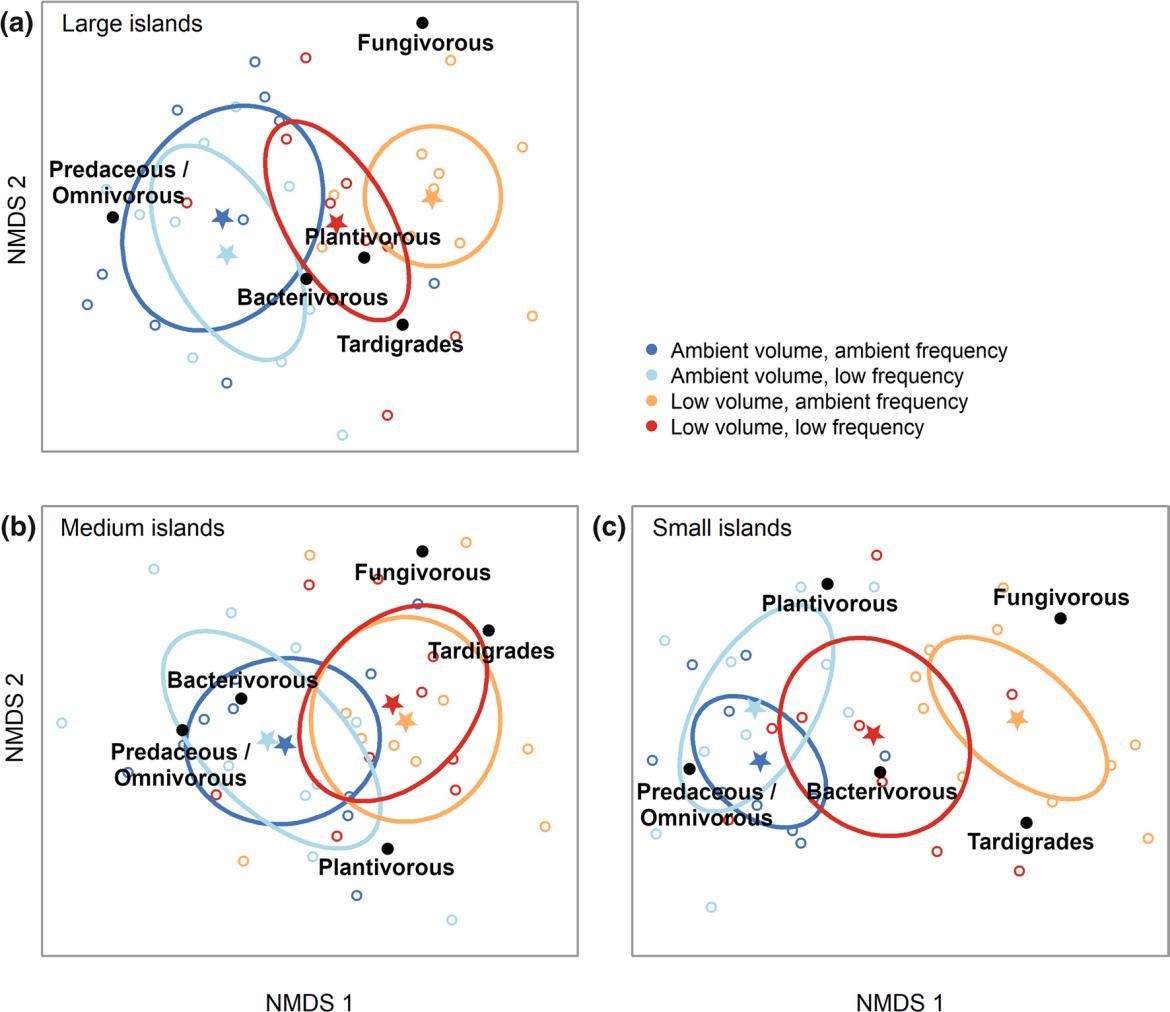
NMDS diagrams of microfauna abundance grouped by faunal functional group, plotted separately for each island size class. Functional groups include nematodes (bacterivorous, plantivorous, predaceous/omnivorous, and fungivorous), and tardigrades. Open circles are samples, stars are centroids of precipitation treatments, and ellipses are standard deviations for the treatment centroids

Island size mediated the effect of precipitation volume on the abundance of total microfauna, all nematodes, and bacterial-feeding nematodes (Table S3), because low volume significantly increased abundance in small islands, but not in medium or large islands (Fig. 2). Island size also mediated the effect of precipitation volume on microfaunal community composition (Table S5), because the effect of volume on community composition was greatest in smaller islands (Fig. 6). Conversely, the response of mesofaunal abundance and community composition to precipitation volume and frequency was unaffected by island size (Table S3, Table S5). Finally, the Shannon diversity of microfaunal functional groups responded to an interactive effect of island size and precipitation frequency, because reduced frequency increased diversity for medium islands, but had no effect for large and small islands (Figure S1, Table S4).

## Discussion

Drought can have major impacts on bryosphere biota and the functions that they perform. Using a mesocosm experiment based on bryospheres and soils collected from a boreal forest chronosequence, we found that precipitation volume and, more weakly, precipitation frequency, impacted the abundance, diversity, and composition of bryosphere micro- and mesofauna. Interactive effects of precipitation volume and frequency were detected for some faunal functional groups, but not for overall abundance. Forest successional stage mediated the effect of precipitation regime on abundance, diversity, and composition of bryosphere microfauna only. Below, we discuss these findings in relation to our hypotheses.

### Drought effects on bryosphere fauna

We found that drier conditions (i.e., low volume and/or frequency of precipitation) increased overall bryosphere fauna abundance, contrary to our first hypothesis and to many studies in bryospheres and soils that have reported negative effects of drought on the abundance of microfauna (Jönsson 2003; Kardol et al. 2010; de Vries et al. 2012) and mesofauna (Lindberg et al. 2002; Makkonen et al. 2011; Lindo et al. 2012). Our findings of neutral (microfauna) and neutral or positive (mesofauna) responses of diversity to drought were also contrary to our first hypothesis. In particular, we expected the abundance and diversity of nematodes and tardigrades to be strongly reduced by drought, given that they live in water films (Coleman et al. 2018a). However, it is possible that moss-associated fauna is adapted to the frequent drying-rewetting cycles that take place in the bryosphere and are therefore resistant to drought (Taylor et al. 2004; Jönsson 2007), for example through anhydrobiosis (Demeure et al. 1979; Nelson 2002). On the other hand, we found that drought impaired predaceous/omnivorous nematodes. This agrees with the theory that nematodes with ‘persistence’ life-history traits (e.g., predaceous/omnivorous nematodes) are most sensitive to disturbances (Bongers 1999; Franco et al. 2017), and is in line with our finding that drought decreased the nematode Maturity Index (i.e., increased the relative abundance of nematodes with a “coloniser” rather than “persister” strategy; Bongers 1990). Therefore, drought may have impaired top-down control of microfauna and led to increased overall microfaunal abundance (Wardle and Yeates 1993; Sylvain et al. 2014; Franco et al. 2019). Additionally, drought impaired springtails, which may have alleviated competition for food with fungivorous and plantivorous nematodes and thus contributed to increasing nematode abundance through competitive release. Taken together, our findings suggest that potential direct negative effects of drought on bryosphere microfauna were overridden by positive drought effects through altered trophic interactions.

Effects of drought were weaker on mesofauna than on microfauna, consistent with our first hypothesis. This is likely due to the greater mobility of mesofauna, which could have more easily avoided drought in the bryosphere by moving to the humus layer, where moisture content is higher and more stable than the in the bryosphere (Lindo et al. 2012; Grau-Andrés et al. 2021b). Drought increased overall mesofaunal abundance mainly through promoting Oribatid mites, which were the most abundant mesofaunal group, consistent with previous bryosphere research (Lindo and Gonzalez 2010; Barreto et al. 2021). The observed increase in mesofaunal diversity with drier conditions has also been previously reported in moss/soil mesocosms (Turnbull and Lindo 2015). We found no evidence that precipitation regime affected mesofaunal functional groups differently, contrary to our hypothesis that predaceous mesofauna (i.e., Mesostigmatid and Prostigmatid mites) would be more sensitive to drought than non-predaceous mesofauna (i.e., Oribatid mites, springtails). Therefore, the observed positive effect of drought on overall mesofaunal abundance cannot be explained by release from predation, because drought also promoted predaceous groups. Some studies have shown negligible effects of drought on soil mite abundance (Kardol et al. 2011; Holmstrup et al. 2013; Sylvain et al. 2014), but positive effects are rare (de Vries et al. 2012), which suggests that the driving mechanisms for the increase in abundance that we found might be bryosphere-specific. One possible explanation is that drying-rewetting cycles that were more extreme promoted food availability for detrivorous Oribatida through damage to moss tissues and subsequent release of intracellular contents (Slate et al. 2019). Springtails were the only mesofaunal functional group impaired by drought, in line with previous work pointing to the drought-sensitivity of springtails (Makkonen et al. 2011; Blankinship et al. 2011). Conversely, the predominantly fungivorous Prostigmatid Tydeidae mites were strongly associated with drier conditions, as has previously been observed in boreal bryospheres (Lindo et al. 2012). This could indicate a drought-induced shift towards a more fungal-based energy channel in the bryosphere, as has been observed in soil (de Vries et al. 2012). Overall, our results support the view that drought affects mesofauna indirectly through changing food resources and trophic interactions (Holmstrup et al. 2013; Wu et al. 2014; Barreto et al. 2021), rather than through direct effects.

### Effects of precipitation volume and frequency

Our second hypothesis predicted stronger effects of reduced precipitation frequency at reduced precipitation volumes because at ambient volumes the wetter humus can alleviate drought intensity in the bryosphere. We found support for this hypothesis for some faunal functional groups, but not for the overall abundance and diversity of microfauna or mesofauna. For predaceous/omnivorous nematodes and for springtails, low-frequency precipitation reduced abundance at low but not at ambient precipitation volumes. Given that these functional groups are known to be particularly sensitive to disturbances, including drought (Blankinship et al. 2011; Franco et al. 2019), our results suggest that frequency and volume only interact to drive the abundance of those micro- and mesofaunal functional groups that are the most responsive to drought. This is in line with our finding that ambient frequency, which led to the driest moss conditions (Fig. 1), reduced the nematode Maturity Index at low but not ambient volumes of precipitation. We also found some evidence that precipitation frequency impacted mesofaunal community composition more at low compared to ambient volumes, in line with our second hypothesis. However, contrary to our expectation, it was ambient rather than low frequency in low volume treatments that shifted the community composition further away from that in ambient precipitation treatments. This interactive effect may have occurred because more water was delivered at the same time in the low frequency than at the ambient frequency treatments, thus minimising evaporative losses and leading to a wetter humus under low precipitation treatments (Soudzilovskaia et al. 2011; Grau-Andrés et al. 2021b), which could have enabled the mesofauna to avoid bryosphere drought.

Precipitation volume had generally stronger effects than precipitation frequency on bryosphere fauna, including on community composition of microfauna and mesofauna, and on the abundance of overall microfauna. This contrasts with previous findings that precipitation frequency had larger or similar effects to volume on bryosphere biota in boreal forests (Jackson et al. 2011). The discrepancy may be due to the inclusion in our experiment of the upper humus layer, which regulates and buffers bryosphere moisture dynamics. Consequently, volume effects dominated because wetter humus at ambient precipitation volumes helped bryospheres remain wet irrespective of frequency. Nevertheless, the abundance of Oribatid mites, and of total mesofauna, responded to precipitation frequency but not volume. This could be due to a combination of two factors: first, moisture levels across both volume levels were within the tolerance range of Oribatid mites (Kardol et al. 2011; Barreto et al. 2021); and second, food supply was enhanced under more frequent drying-rewetting cycles (Slate et al. 2019). In total, our results suggest that functional group identity may determine whether the main effects of volume and/or frequency, as well as their interactive effects, drive the response of bryosphere fauna to changing precipitation regimes.

### Context-dependency of drought effects

We found that drought effects on bryosphere fauna abundance and composition depended on the boreal forest successional stage for microfauna, but not mesofauna. Drought altered microfauna community composition and nematode abundance in late-successional forests, but not in mid- or early-successional forests. This is contrary to our third hypothesis predicting that drought effects would be greatest in early-successional forests on the basis that the more bacterial-based microbial food webs of those forests would be more sensitive to drought than would the more fungal-based food webs of late-successional forests. Given the little evidence we found of direct negative effects of drought on micro- or mesofaunal abundance, the faunal patterns we observed across the succession gradient cannot be explained by impairment of microbial communities by drought, which suggests that other mechanisms prevailed, such as changes in food sources or trophic interactions. One possibility is that variation in nutrient availability along the gradient drove the response of bryosphere fauna to drought. While in our study system the levels of available soil nutrients are lowest in late-successional forests (Wardle et al. 2003, 2012), there is also greater N-fixation by symbiotic cyanobacteria inhabiting the bryosphere in late-successional forests (Lagerström et al. 2007) and thus greater N in moss tissues (Bansal et al. 2012). Therefore, nitrogen availability for bryosphere fauna may have been greater in late- compared to early-successional forests (Kardol et al. 2016). Consequently, a less nutrient-limited bryosphere fauna in late-successional forests may have resulted in a greater impairment of top-down control following drought in late- compared to early-successional forests. Our findings agree with the theory for soil food webs that top-down control is greatest in the absence of bottom-up limitation (Crowther et al. 2015), and point to context-dependent effects of drought on bryosphere microfauna.

## Conclusions

The observed increase in bryosphere faunal abundance following reduced precipitation volume or frequency suggests that fauna-induced enhancement of carbon and nutrient turnover in boreal forest bryospheres may increase as a result of drought. The subsequent increase in nutrient supply could help compensate for the expected decrease in ecosystem productivity from drought. However, the reduced bryosphere carbon cycling and moss growth under drought that we observed in the same experiment indicates that drought overall impairs bryosphere productivity (Grau-Andrés et al. 2021b). Further, we found that precipitation volume generally had stronger effects than did precipitation frequency on bryosphere fauna, likely because moisture supply from the lower bryosphere and upper humus layers buffered any adverse effects of reduced precipitation frequency. As climate projections for the boreal forest biome show that drought will be mainly driven by more infrequent precipitation (Collins et al. 2013), bryosphere fauna may be relatively resilient to drought for thicker and denser bryospheres and those on thicker humus layers because of their higher water holding capacity (Elumeeva et al. 2011; Grau-Andrés et al. 2021b). Finally, our findings point to a greater vulnerability of bryosphere fauna to altered precipitation regimes in late- compared to early-successional forests. Given that bryospheres dominated by different moss species can host different micro- and mesofaunal communities (Jonsson et al. 2015; Grau-Andrés et al. 2021a), future work should assess whether our findings for *Hylocomium splendens* bryospheres can be generalised to other common boreal bryospheres (e.g., *Pleurozium schreberi*). The bryosphere plays an important role in key boreal forest functions including ecosystem productivity and nitrogen supply, especially in older forests (Gundale et al. 2009; Wardle et al. 2012). As those functions can be affected by bryosphere fauna (Schill et al. 2011; Kardol et al. 2016), our results suggest that the effects of altered precipitation regimes on boreal forest functioning could be mediated by changes in bryosphere fauna and that these effects may be stronger in older forests.

## Supplementary Information

Below is the link to the electronic supplementary material.

Supplementary file1 (DOCX 121 KB)

## Data Availability

Data underpinning this research are openly available in figshare at https://doi.org/10.6084/m9.figshare.19626438.v1

## References

[CR1] Anderson MJ (2001). A new method for non-parametric multivariate analysis of variance: non-parametric MANOVA for ecology. Austral Ecol.

[CR2] Bansal S, Nilsson M-C, Wardle DA (2012). Response of photosynthetic carbon gain to ecosystem retrogression of vascular plants and mosses in the boreal forest. Oecologia.

[CR3] Bardgett RD, van der Putten WH (2014). Belowground biodiversity and ecosystem functioning. Nature.

[CR4] Barreto C, Lindo Z (2018). Drivers of decomposition and the detrital invertebrate community differ across a hummock-hollow microtopology in Boreal peatlands. Écoscience.

[CR5] Barreto C, Branfireun BA, McLaughlin J, Lindo Z (2021). Responses of oribatid mites to warming in boreal peatlands depend on fen type. Pedobiologia.

[CR6] Berg MP, Kniese JP, Bedaux JJM, Verhoef HA (1998). Dynamics and stratification of functional groups of micro- and mesoarthropods in the organic layer of a Scots pine forest. Biol Fertil Soils.

[CR7] Berg A, Sheffield J, Milly PCD (2017). Divergent surface and total soil moisture projections under global warming: future soil moisture changes in coupled model intercomparison project phase 5. Geophys Res Lett.

[CR8] Blankinship JC, Niklaus PA, Hungate BA (2011). A meta-analysis of responses of soil biota to global change. Oecologia.

[CR9] Bokhorst S, Wardle DA, Nilsson M-C, Gundale MJ (2014). Impact of understory mosses and dwarf shrubs on soil micro-arthropods in a boreal forest chronosequence. Plant Soil.

[CR10] Bongers T (1990). The maturity index: an ecological measure of environmental disturbance based on nematode species composition. Oecologia.

[CR11] Bongers T (1999). The Maturity Index, the evolution of nematode life history traits, adaptive radiation and cp-scaling. Plant Soil.

[CR12] Bongers T, Ferris H (1999). Nematode community structure as a bioindicator in environmental monitoring. Trends Ecol Evol.

[CR13] Bongers T (1988) De nematoden van Nederland: een identificatietabel voor de in Nederland aangetroffen zoetwater-en bodembewonende nematoden. Stichting Uitgeverij Koninklijke Nederlandse Natuurhistorische Vereniging

[CR14] Carleton TJ, Dunham KMM (2003). Distillation in a boreal mossy forest floor. Can J for Res.

[CR15] Clemmensen KE, Bahr A, Ovaskainen O (2013). Roots and associated fungi drive long-term carbon sequestration in boreal forest. Science.

[CR16] Clemmensen KE, Finlay RD, Dahlberg A (2015). Carbon sequestration is related to mycorrhizal fungal community shifts during long-term succession in boreal forests. New Phytol.

[CR17] Coleman DC, Callaham MA, Crossley DA (2018). Laboratory and field exercises in soil ecology. Fundamentals of soil ecology.

[CR18] Coleman DC, Callaham MA, Crossley DA (2018a) Secondary Production. In: Fundamentals of Soil Ecology. Elsevier, pp 77–171

[CR19] Collins A, Knutti R, Arblaster J, Stocker TF, Qin D (2013). Long-term climate change: projections, commitments and irreversibility. Climate change 2013—the physical science basis: contribution of working group i to the fifth assessment report of the intergovernmental panel on climate change.

[CR20] Crowther TW, Thomas SM, Maynard DS (2015). Biotic interactions mediate soil microbial feedbacks to climate change. Proc Natl Acad Sci USA.

[CR21] de Vries FT, Liiri ME, Bjørnlund L (2012). Land use alters the resistance and resilience of soil food webs to drought. Nat Clim Chang.

[CR22] de Vries FT, Thébault E, Liiri M (2013). Soil food web properties explain ecosystem services across European land use systems. Proc Natl Acad Sci USA.

[CR23] Delgado-Baquerizo M, Reich PB, Trivedi C (2020). Multiple elements of soil biodiversity drive ecosystem functions across biomes. Nat Ecol Evol.

[CR24] DeLucia EH, Turnbull MH, Walcroft AS (2003). The contribution of bryophytes to the carbon exchange for a temperate rainforest. Glob Change Biol.

[CR25] Demeure Y, Freckman DW, Van Gundy SD (1979). Anhydrobiotic coiling of nematodes in soil. J Nematol.

[CR26] Elumeeva TG, Soudzilovskaia NA, During HJ, Cornelissen JHC (2011). The importance of colony structure versus shoot morphology for the water balance of 22 subarctic bryophyte species: factors affecting bryophyte water balance. J Veg Sci.

[CR27] Faith DP, Minchin PR, Belbin L (1987). Compositional dissimilarity as a robust measure of ecological distance. Vegetatio.

[CR28] Fanin N, Gundale MJ, Farrell M (2018). Consistent effects of biodiversity loss on multifunctionality across contrasting ecosystems. Nat Ecol Evol.

[CR29] Ferris H (2010). Contribution of nematodes to the structure and function of the soil food web. J Nematol.

[CR30] Filser J (2002). The role of Collembola in carbon and nitrogen cycling in soil. Pedobiologia.

[CR31] Franco ALC, Knox MA, Andriuzzi WS (2017). Nematode exclusion and recovery in experimental soil microcosms. Soil Biol Biochem.

[CR32] Franco ALC, Gherardi LA, de Tomasel CM (2019). Drought suppresses soil predators and promotes root herbivores in mesic, but not in xeric grasslands. Proc Natl Acad Sci USA.

[CR33] Gauthier S, Bernier P, Kuuluvainen T (2015). Boreal forest health and global change. Science.

[CR34] Grau-Andrés R, Wardle DA, Gundale MJ (2020). Effects of plant functional group removal on CO _2_ fluxes and belowground C stocks across contrasting ecosystems. Ecology.

[CR35] Grau-Andrés R, Wardle DA, Kardol P (2021). Bryosphere loss impairs litter decomposition consistently across moss species, litter types, and micro-arthropod abundance. Ecosystems.

[CR36] Grau-Andrés R, Wardle DA, Nilsson M-C, Kardol P (2021). Precipitation regime controls bryosphere carbon cycling similarly across contrasting ecosystems. Oikos.

[CR37] Gundale MJ, Gustafsson H, Nilsson M-C (2009). The sensitivity of nitrogen fixation by a feathermoss-cyanobacteria association to litter and moisture variability in young and old boreal forests. Can J for Res.

[CR38] Hansen MC, Stehman SV, Potapov PV (2010). Quantification of global gross forest cover loss. Proc Natl Acad Sci USA.

[CR39] Heidemann K, Ruess L, Scheu S, Maraun M (2014). Nematode consumption by mite communities varies in different forest microhabitats as indicated by molecular gut content analysis. Exp Appl Acarol.

[CR40] Heijmans MMPD, Arp WJ, Chapin FS (2004). Carbon dioxide and water vapour exchange from understory species in boreal forest. Agric for Meteorol.

[CR41] Holmstrup M, Sørensen JG, Schmidt IK (2013). Soil microarthropods are only weakly impacted after 13 years of repeated drought treatment in wet and dry heathland soils. Soil Biol Biochem.

[CR42] Holmstrup M, Damgaard C, Schmidt IK (2017). Long-term and realistic global change manipulations had low impact on diversity of soil biota in temperate heathland. Sci Rep.

[CR43] Holtkamp R, Kardol P, van der Wal A (2008). Soil food web structure during ecosystem development after land abandonment. Appl Soil Ecol.

[CR44] Ingham RE, Trofymow JA, Ingham ER, Coleman DC (1985). Interactions of bacteria, fungi, and their nematode grazers: effects on nutrient cycling and plant growth. Ecol Monogr.

[CR45] Jackson BG, Martin P, Nilsson M-C, Wardle DA (2011). Response of feather moss associated N_2_ fixation and litter decomposition to variations in simulated rainfall intensity and frequency. Oikos.

[CR46] Jackson BG, Nilsson M-C, Wardle DA (2013). The effects of the moss layer on the decomposition of intercepted vascular plant litter across a post-fire boreal forest chronosequence. Plant Soil.

[CR47] Jairajpuri MS, Ahmad W (1992) Dorylaimida: free-living, predaceous and plant-parasitic nematodes. Brill

[CR48] Jönsson KI (2003). Population density and species composition of moss-living tardigrades in a boreo-nemoral forest. Ecography.

[CR49] Jönsson KI (2007). Long-term experimental manipulation of moisture conditions and its impact on moss-living tardigrades. J Limnol.

[CR50] Jonsson M, Kardol P, Gundale MJ (2015). Direct and indirect drivers of moss community structure, function, and associated microfauna across a successional gradient. Ecosystems.

[CR51] Kamath D, Barreto C, Lindo Z (2022). Nematode contributions to the soil food web trophic structure of two contrasting boreal peatlands in Canada. Pedobiologia.

[CR52] Kardol P, Cregger MA, Campany CE, Classen AT (2010). Soil ecosystem functioning under climate change: plant species and community effects. Ecology.

[CR53] Kardol P, Reynolds WN, Norby RJ, Classen AT (2011). Climate change effects on soil microarthropod abundance and community structure. Appl Soil Ecol.

[CR54] Kardol P, Spitzer CM, Gundale MJ (2016). Trophic cascades in the bryosphere: the impact of global change factors on top-down control of cyanobacterial N_2_-fixation. Ecol Lett.

[CR55] Kardol P, Fanin N, Wardle DA (2018). Long-term effects of species loss on community properties across contrasting ecosystems. Nature.

[CR56] Krantz GW, Walter DE (2009). A manual of Acarology.

[CR57] Kumordzi BB, Wardle DA, Freschet GT (2015). Plant assemblages do not respond homogenously to local variation in environmental conditions: functional responses differ with species identity and abundance. J Veg Sci.

[CR58] Lagerström A, Nilsson M-C, Zackrisson O, Wardle DA (2007). Ecosystem input of nitrogen through biological fixation in feather mosses during ecosystem retrogression. Funct Ecol.

[CR59] Lagerström A, Nilsson M-C, Wardle DA (2013). Decoupled responses of tree and shrub leaf and litter trait values to ecosystem retrogression across an island area gradient. Plant Soil.

[CR60] Lenth R (2021) emmeans: estimated marginal means, aka least-squares means. Version 1.6.3. URL: https://CRAN.R-project.org/package=emmeans

[CR61] Lindberg N, Engtsson JB, Persson T (2002). Effects of experimental irrigation and drought on the composition and diversity of soil fauna in a coniferous stand: Long-term drought affects soil animal diversity. J Appl Ecol.

[CR62] Lindo Z, Gonzalez A (2010). The bryosphere: an integral and influential component of the earth’s biosphere. Ecosystems.

[CR63] Lindo Z, Whiteley J, Gonzalez A (2012). Traits explain community disassembly and trophic contraction following experimental environmental change. Glob Change Biol.

[CR64] Lindo Z, Nilsson M-C, Gundale MJ (2013). Bryophyte-cyanobacteria associations as regulators of the northern latitude carbon balance in response to global change. Glob Change Biol.

[CR65] Makkonen M, Berg MP, van Hal JR (2011). Traits explain the responses of a sub-arctic Collembola community to climate manipulation. Soil Biol Biochem.

[CR66] Nelson DR (2002). Current status of the tardigrada: evolution and ecology. Integr Comp Biol.

[CR67] Oksanen J, Blanchet FG, Friendly M, et al (2020) vegan: community ecology package. Version 2.5–7. URL: https://CRAN.R-project.org/package=vegan

[CR68] Pinheiro J, Bates D, DebRoy S, Sarkar D (2021) nlme: linear and nonlinear mixed effects models. Version 3.1–152. URL: https://CRAN.R-project.org/package=nlme

[CR69] R Core Team (2021) R: a language and environment for statistical computing. R Foundation for Statistical Computing

[CR70] Sackett TE, Classen AT, Sanders NJ (2010). Linking soil food web structure to above- and belowground ecosystem processes: a meta-analysis. Oikos.

[CR71] Schill RO, Jönsson KI, Pfannkuchen M, Brümmer F (2011). Food of tardigrades: a case study to understand food choice, intake and digestion. J Zool Syst Evol Res.

[CR72] Seastedt T (1984). The role of microarthropods in decomposition and mineralization processes. Annu Rev Entomol.

[CR73] Slate ML, Sullivan BW, Callaway RM (2019). Desiccation and rehydration of mosses greatly increases resource fluxes that alter soil carbon and nitrogen cycling. J Ecol.

[CR74] Soil Survey Staff (2015) Illustrated guide to soil taxonomy. Department of Agriculture, Natural Resources Conservation Service, National Soil Survey Center, Lincoln, Nebraska

[CR75] Soudzilovskaia NA, Graae BJ, Douma JC (2011). How do bryophytes govern generative recruitment of vascular plants?. New Phytol.

[CR76] Street LE, Subke J-A, Sommerkorn M (2013). The role of mosses in carbon uptake and partitioning in arctic vegetation. New Phytol.

[CR77] Sylvain ZA, Wall DH, Cherwin KL (2014). Soil animal responses to moisture availability are largely scale, not ecosystem dependent: insight from a cross-site study. Glob Change Biol.

[CR78] Taylor AR, Schröter D, Pflug A, Wolters V (2004). Response of different decomposer communities to the manipulation of moisture availability: potential effects of changing precipitation patterns: Litter Moisture and Decomposer Communities. Glob Change Biol.

[CR79] Turetsky MR, Mack MC, Hollingsworth TN, Harden JW (2010). The role of mosses in ecosystem succession and function in Alaska’s boreal forest. Can J for Res.

[CR80] Turnbull MS, Lindo Z (2015). Combined effects of abiotic factors on Collembola communities reveal precipitation may act as a disturbance. Soil Biol Biochem.

[CR81] van den Hoogen J, Geisen S, Routh D (2019). Soil nematode abundance and functional group composition at a global scale. Nature.

[CR82] Wagg C, Bender SF, Widmer F, van der Heijden MGA (2014). Soil biodiversity and soil community composition determine ecosystem multifunctionality. Proc Natl Acad Sci USA.

[CR83] Wall DH, Bradford MA, St. John MG (2008). Global decomposition experiment shows soil animal impacts on decomposition are climate-dependent. Glob Change Biol.

[CR84] Walter DE, Proctor HC, Walter DE, Proctor HC (2013). Mites in soil and litter systems. Mites: ecology, evolution & behaviour: life at a microscale.

[CR85] Wardle D, Yeates G (1993). The dual importance of competition and predation as regulatory forces in terrestrial ecosystems: evidence from decomposer food-webs. Oecologia.

[CR86] Wardle DA, Zackrisson O, Hörnberg G, Gallet C (1997). The influence of island area on ecosystem properties. Science.

[CR87] Wardle DA, Hörnberg G, Zackrisson O (2003). Long-term effects of wildfire on ecosystem properties across an island area gradient. Science.

[CR88] Wardle DA, Bardgett RD, Klironomos JN (2004). Ecological linkages between aboveground and belowground biota. Science.

[CR89] Wardle DA, Jonsson M, Bansal S (2012). Linking vegetation change, carbon sequestration and biodiversity: insights from island ecosystems in a long-term natural experiment. J Ecol.

[CR90] Wu T, Su F, Han H (2014). Responses of soil microarthropods to warming and increased precipitation in a semiarid temperate steppe. Appl Soil Ecol.

[CR91] Yeates GW, Bongers T, De Goede RG (1993). Feeding habits in soil nematode families and genera-an outline for soil ecologists. J Nematol.

[CR92] Yin R, Eisenhauer N, Auge H (2019). Additive effects of experimental climate change and land use on faunal contribution to litter decomposition. Soil Biol Biochem.

[CR93] Yin R, Gruss I, Eisenhauer N (2019). Land use modulates the effects of climate change on density but not community composition of Collembola. Soil Biol Biochem.

[CR94] Zuur A, Ieno EN, Walker N (2009). Mixed effects models and extensions in ecology with R.

