# Supplementary material for: Responses of bryosphere fauna to drought across a boreal forest chronosequence
Source: Oecologia. 2022 Sep 8;200(1-2):231–45. doi: 10.1007/s00442-022-05255-z (PMC9547781; doi:10.1007/s00442-022-05255-z)
Supplement: Supplementary file 1 — Supplementary file1 (DOCX 121 KB) [file 442_2022_5255_MOESM1_ESM.docx]

# Supporting Information

**Table S1.** Variability in time since last wildfire and ecosystem properties (mean ± SE) across island size classes. Data from Wardle et al. 1997, 2003, 2012, Lagerström et al. 2009 and Clemmensen et al. 2013. Within each row, numbers followed by the same letter are not significantly different (i.e., P > 0.05) according to Tukey’s tests.

| Ecosystem properties |  | Island size |  |
| --- | --- | --- | --- |
|  | Small | Medium | Large |
| Time since last fire (years) | 3250 ± 439 a | 2180 ± 385 b | 585 ± 233 c |
| Net primary productivity (g m^-2^ yr^-1^) | 159 ± 18 b | 247 ± 12 a | 256 ± 14 a |
| Standing plant biomass (g m^-2^) | 3470 ± 470 b | 8340 ± 877 a | 9349 ± 485 a |
| Vascular plant species richness | 10.6 ± 0.6 a | 8.6 ± 0.4 b | 6.6 ± 0.6 c |
| Humus C to N ratio | 32.9 ± 0.79 b | 36.0 ± 1.17 ab | 40.4 ± 1.18 a |
| Humus C to P ratio | 759 ± 30 a | 687 ± 36 ab | 623 ± 20 b |
| Humus N to P ratio | 23.3 ± 1.1 a | 19.1 ± 0.9 b | 15.4 ± 0.5 c |
| Mineral N (MIN) (*µ*g N g^-1^) | 25.3 ± 8.0 b | 58.1 ± 9.2 a | 38.2 ± 14.4 ab |
| Dissolved organic N (DON) (*µ*g N g^-1^) | 40.3 ± 4.6 b | 50.7 ± 5.5 a | 39.1 ± 7.2 b |
| MIN/(MIN+DON) | 0.39 ± 0.03 b | 0.53 ± 0.05 a | 0.49 ± 0.04 a |
| Mineral P (*µ*g P g^-1^) | 24.4 ± 2.3 b | 37.7 ± 4.3 a | 43.6 ± 4.9 a |
| Membrane-extractable P (mmol kg^-1^) | 4.9 ± 0.3 b | 6.5 ± 0.4 a | 5.9 ± 0.7 ab |
| Light transmission (%) | 68.6 ± 2.6 a | 47.1 ± 3.7 b | 55.8 ± 4.5 ab |

**Table S2.** Mean (range) abundance of nematode taxa (individuals g^-1^ dry moss) across precipitation regimes (volume (vol), frequency (fre)) and island size classes. The precipitation treatments resulted from the combination of two levels (Ambient and Low) of precipitation volume and frequency. Feeding group (Pred./omn. = Predaceous / omnivorous) is based on Yeates et al. (1993). The colonizer–persister score (c–p) is based on Bongers (1990).

|  |  |  |  | Water addition regime | | | |  | Island size class | | |
| --- | --- | --- | --- | --- | --- | --- | --- | --- | --- | --- | --- |
| Nematode taxa | Feeding group | c–p |  | A vol - A fre | A vol - L fre | L vol - A fre | L vol - L fre |  | Large | Medium | Small |
| Alaimidae | Bacterivorous | 4 |  | 0.4 (0-8) | 1.4 (0-13) | 0.4 (0-13) | 4.6 (0-77) |  | 1.2 (0-13) | 2.5 (0-77) | 1.5 (0-28) |
| Anguinidae | Fungivorous | 2 |  | 12.3 (0-129) | 2.8 (0-36) | 11 (0-100) | 5.6 (0-62) |  | 8.8 (0-72) | 9.3 (0-129) | 5.7 (0-62) |
| Aphelenchoididae | Fungivorous | 2 |  | 17.8 (0-102) | 12.1 (0-75) | 132.7 (0-839) | 25.1 (0-184) |  | 30 (0-184) | 38.2 (0-620) | 72.7 (0-839) |
| Bunonematodae | Bacterivorous | 1 |  | 17.6 (0-82) | 32.1 (0-385) | 50.6 (0-298) | 34.2 (0-236) |  | 29.7 (0-385) | 27.9 (0-236) | 43.3 (0-298) |
| Cephalobidae | Bacterivorous | 2 |  | 31.4 (0-549) | 18.6 (0-106) | 165.5 (2-1321) | 77.9 (7-267) |  | 35.9 (0-231) | 77.7 (0-549) | 106.4 (0-1321) |
| Desmodoridae | Bacterivorous | 3 |  | 129.3 (0-549) | 114.3 (0-473) | 138 (0-908) | 158.5 (11-733) |  | 101.9 (0-346) | 157.8 (0-549) | 145.4 (0-908) |
| Dorylaimoidea | Pred./omn. | 4 |  | 205.1 (12-920) | 155.3 (0-482) | 92.8 (0-439) | 114.3 (5-472) |  | 139.5 (0-482) | 168.6 (1-920) | 117.5 (0-406) |
| Monhysteridae | Bacterivorous | 1 |  | 63.4 (0-406) | 50.5 (0-941) | 38.6 (0-384) | 7.5 (0-44) |  | 21.6 (0-234) | 66.9 (0-941) | 31.6 (0-384) |
| Mononchidae | Pred./omn. | 4 |  | 26.5 (0-223) | 25.8 (0-217) | 3.3 (0-30) | 7.8 (0-83) |  | 17 (0-223) | 7.6 (0-45) | 23 (0-217) |
| Plectidae | Bacterivorous | 2 |  | 287.9 (29-1612) | 193.1 (34-683) | 398.7 (17-2338) | 241 (78-917) |  | 229.5 (29-711) | 301.4 (17-1612) | 309.6 (29-2338) |
| Prismatolamidae | Bacterivorous | 3 |  | 97.6 (0-423) | 80.1 (0-288) | 7.5 (0-84) | 9.5 (0-105) |  | 61.6 (0-423) | 60.2 (0-352) | 24.2 (0-222) |
| Rhabditidae | Bacterivorous | 1 |  | 0 (0-0) | 0.5 (0-8) | 0 (0-0) | 0 (0-0) |  | 0.2 (0-7) | 0.2 (0-8) | 0 (0-0) |
| Teratocephalidae | Bacterivorous | 3 |  | 140 (2-789) | 205.8 (23-1083) | 333.4 (12-1275) | 366.2 (69-1205) |  | 229.2 (2-1237) | 248.3 (12-1205) | 306.7 (23-1275) |
| Tripylidae | Pred./omn. | 3 |  | 2 (0-28) | 4.6 (0-102) | 19.3 (0-223) | 28.2 (0-140) |  | 4.9 (0-77) | 17 (0-223) | 18.7 (0-140) |
| Tylenchidae | Plantivorous | 2 |  | 210.3 (19-848) | 208.4 (4-677) | 347.9 (5-1490) | 322.5 (52-998) |  | 249.3 (5-1490) | 322.6 (4-848) | 245 (11-1275) |

**Table S3.** Results of linear mixed effects models testing the response of abundance of microfauna to variation in volume and frequency of precipitation, and island size class. numDF and denDF are numerator and denominator degrees of freedom. Significant effects at α = 0.05 are bolded. R^2^m is R^2^ marginal and R^2^c is R^2^ conditional.

|  |  |  | All  microfauna | All  nematodes | Tardigrades | Nematode feeding groups | | | |
| --- | --- | --- | --- | --- | --- | --- | --- | --- | --- |
|  |  |  |  |  |  | Bacterivorous | Plantivorous | Predaceous /  omnivorous | Fungivorous |
|  | NumDF | DenDF | *F* (*P*) | *F* (*P*) | *F* (*P*) | *F* (*P*) | *F* (*P*) | *F* (*P*) | *F* (*P*) |
| Volume (V) | 1 | 81 | **19.7 (<0.001)** | **6.7 (0.011)** | **51.7 (<0.001)** | **12.6 (0.001)** | **11.5 (0.001)** | **11.1 (0.001)** | **17.2 (<0.001)** |
| Frequency (F) | 1 | 81 | 0.1 (0.752) | 0.3 (0.617) | 0.3 (0.588) | 3.4 (0.071) | 1.7 (0.199) | 0.1 (0.776) | 3.7 (0.058) |
| Island size (IS) | 2 | 27 | **3.6 (0.041)** | 1.7 (0.21) | 1.8 (0.187) | 1.2 (0.305) | 1.5 (0.241) | 0.1 (0.909) | 0.1 (0.917) |
| V × F | 1 | 81 | 0.7 (0.417) | 0.6 (0.442) | 0 (0.956) | 0 (0.908) | 0.7 (0.414) | **8.6 (0.004)** | 1 (0.311) |
| V × IS | 2 | 81 | **3.7 (0.030)** | **3.1 (0.050)** | 1.4 (0.245) | **5.1 (0.008)** | 2.2 (0.118) | 0.2 (0.837) | 0.7 (0.509) |
| F × IS | 2 | 81 | 0.2 (0.791) | 0.4 (0.686) | 0 (0.953) | 0.2 (0.794) | 2.3 (0.104) | 1.6 (0.205) | 0.6 (0.533) |
| V × F × IS | 2 | 81 | 0.1 (0.885) | 0.1 (0.904) | 0.2 (0.850) | 0.1 (0.898) | 0.2 (0.833) | 0.5 (0.601) | 0.6 (0.560) |
| R^2^m, R^2^c |  |  | 0.15, 0.15 | 0.09, 0.12 | 0.28, 0.28 | 0.11, 0.14 | 0.17, 0.29 | 0.45, 0.52 | 0.12, 0.13 |

**Table S4.** Results of linear mixed effects models testing the response of alpha- and Shannon-diversity to variation in volume and frequency of precipitation, and island size class, separately for nematode taxa, microfaunal functional groups, and mesofaunal functional groups. numDF and denDF are numerator and denominator degrees of freedom. Significant effects at α = 0.05 are bolded. R^2^m is R^2^ marginal and R^2^c is R^2^ conditional.

|  | Nematode taxa | | Microfauna | | Mesofauna | |
| --- | --- | --- | --- | --- | --- | --- |
|  | α-diversity | Shannon | α-diversity | Shannon | α-diversity | Shannon |
|  | *F* (*P*) | *F* (*P*) | *F* (*P*) | *F* (*P*) | *F* (*P*) | *F* (*P*) |
| Volume (V) | 0.0 (0.997) | 1.2 (0.284) | **9.4 (0.003)** | 2.4 (0.122) | 0.0 (0.950) | **6.4 (0.013)** |
| Frequency (F) | 2.2 (0.144) | 0.1 (0.786) | 0.1 (0.814) | 2.2 (0.143) | 2.2 (0.144) | 1.7 (0.199) |
| Island size (IS) | 0.5 (0.590) | 0.3 (0.750) | 0.0 (0.963) | 2.2 (0.129) | 0.4 (0.688) | 2.4 (0.107) |
| V × F | 1.1 (0.304) | 1.0 (0.317) | 0.5 (0.480) | 0.1 (0.750) | 3.3 (0.072) | 1.9 (0.170) |
| V × IS | 0.2 (0.788) | 1.3 (0.269) | 0.4 (0.677) | 2.7 (0.070) | 0.2 (0.847) | 0.1 (0.918) |
| F × IS | 0.1 (0.939) | 0.8 (0.449) | 0.1 (0.946) | **4.1 (0.020)** | 0.2 (0.859) | 0.1 (0.918) |
| V × F × IS | 1.0 (0.369) | 0.5 (0.621) | 0.2 (0.846) | 0.4 (0.646) | 0.0 (0.973) | 0.4 (0.689) |
| R^2^m, R^2^c | 0.04, 0.06 | 0.06, 0.13 | 0.08, 0.18 | 0.25, 0.25 | 0.06, 0.06 | 0.12, 0.14 |

**Table S5.** Results of PERMANOVA testing the response to volume and frequency of precipitation and island size class of microfaunal community composition (grouped by functional groups, i.e., bacterivorous nematodes, fungivorous nematodes, predaceous and omnivorous nematodes, herbivorous nematodes, and tardigrades) and of mesofaunal community composition (grouped by functional groups, i.e., Oribatid mites, Mesostigmatid mites, Prostigmatid mites belonging to the Tydeidae family, other Prostigmatid mites, and springtails).

|  |  | Microfauna | | |  | Mesofauna | | |
| --- | --- | --- | --- | --- | --- | --- | --- | --- |
|  | DF | R^2^ | *F* | *P* |  | R^2^ | *F* | *P* |
| Volume (V) | 1 | 0.076 | **10.1** | **0.001** |  | 0.101 | **13.7** | **0.001** |
| Frequency (F) | 1 | 0.020 | **2.7** | **0.007** |  | 0.034 | **4.6** | **0.001** |
| Island size (IS) | 2 | 0.018 | **1.2** | **0.001** |  | 0.016 | **1.1** | **0.001** |
| V × F | 1 | 0.011 | 1.5 | 0.145 |  | 0.014 | 2.0 | 0.053 |
| V × IS | 2 | 0.033 | **2.2** | **0.012** |  | 0.017 | 1.2 | 0.248 |
| F × IS | 2 | 0.015 | 1.0 | 0.378 |  | 0.011 | 0.8 | 0.620 |
| V × F × IS | 2 | 0.010 | 0.7 | 0.787 |  | 0.011 | 0.8 | 0.632 |
| Residual | 108 | 0.816 |  |  |  | 0.795 |  |  |
| Total | 119 | 1 |  |  |  | 1 |  |  |

**Table S6.** Results of linear mixed effects models testing the response of abundance of mesofauna to variation in volume and frequency of precipitation, and island size class. numDF and denDF are numerator and denominator degrees of freedom. Significant effects at α = 0.05 are bolded. R^2^m is R^2^ marginal and R^2^c is R^2^ conditional.

|  |  |  | All  mesofauna | Springtails |  | Mites | | | | |
| --- | --- | --- | --- | --- | --- | --- | --- | --- | --- | --- |
|  |  |  |  |  | All | Oribatida | Mesostigmata | Prostigmata  (Tydeidae) | Prostigmata  (other) | Unknown  juveniles |
|  | numDF | denDF | *F* (*P*) | *F* (*P*) | *F* (*P*) | *F* (*P*) | *F* (*P*) | *F* (*P*) | *F* (*P*) | *F* (*P*) |
| Volume (V) | 1 | 81 | 0.2 (0.644) | **12.8 (0.001)** | 0.2 (0.698) | 2.1 (0.155) | **18.5 (<0.0001)** | **169.6 (<0.0001)** | 0.6 (0.455) | **5.8 (0.018)** |
| Frequency (F) | 1 | 81 | **6.4 (0.014)** | 3.9 (0.051) | **6.4 (0.013)** | **9.2 (0.003)** | **5.4 (0.022)** | 0.0 (0.914) | 2.4 (0.123) | 1.9 (0.176) |
| Island size (IS) | 2 | 27 | 0.1 (0.915) | 0.1 (0.908) | 0.3 (0.758) | 0.9 (0.438) | 2.2 (0.134) | 1.2 (0.322) | 1.7 (0.210) | 0.2 (0.828) |
| V × F | 1 | 81 | 1.3 (0.257) | **12.4 (0.001)** | 0.1 (0.776) | 0.7 (0.414) | 3.0 (0.088) | **5.4 (0.022)** | 1.0 (0.327) | 1.2 (0.285) |
| V × IS | 2 | 81 | 2.1 (0.128) | 0.1 (0.903) | 1.8 (0.166) | 1.3 (0.266) | 0.2 (0.78) | 2.8 (0.066) | 0.3 (0.754) | 1.4 (0.243) |
| F × IS | 2 | 81 | 0.9 (0.396) | 0.0 (0.989) | 1.1 (0.345) | 0.7 (0.483) | 0.8 (0.44) | 1.5 (0.223) | 0.2 (0.850) | 0.3 (0.752) |
| V × F × IS | 2 | 81 | 0.0 (0.975) | 0.6 (0.532) | 0.2 (0.780) | 0.2 (0.795) | 0.7 (0.49) | 0.2 (0.811) | 0.1 (0.925) | 1.6 (0.215) |
| R^2^m, R^2^c |  |  | 0.08, 0.14 | 0.21, 0.41 | 0.06, 0.11 | 0.08, 0.14 | 0.30, 0.31 | 0.55, 0.56 | 0.08, 0.30 | 0.10, 0.40 |

**Table S7.** Results of a linear mixed effects model testing the response of the Maturity Index (based on the ‘c–p’ framework of Bongers 1990) to variation in volume and frequency of precipitation, and island size class. numDF and denDF are numerator and denominator degrees of freedom. Significant effects at α = 0.05 are bolded. R^2^ marginal was 0.28 and R^2^ conditional was 0.40.

|  | numDF | denDF | *F* (*P*) |
| --- | --- | --- | --- |
| Volume (V) | 1 | 81 | **31.9 (<0.001)** |
| Frequency (F) | 1 | 81 | 3.9 (0.051) |
| Island size (IS) | 2 | 27 | 1.4 (0.272) |
| V × F | 1 | 81 | **6.2 (0.015)** |
| V × IS | 2 | 81 | 1.9 (0.154) |
| F × IS | 2 | 81 | 0.1 (0.867) |
| V × F × IS | 2 | 81 | 2.6 (0.084) |


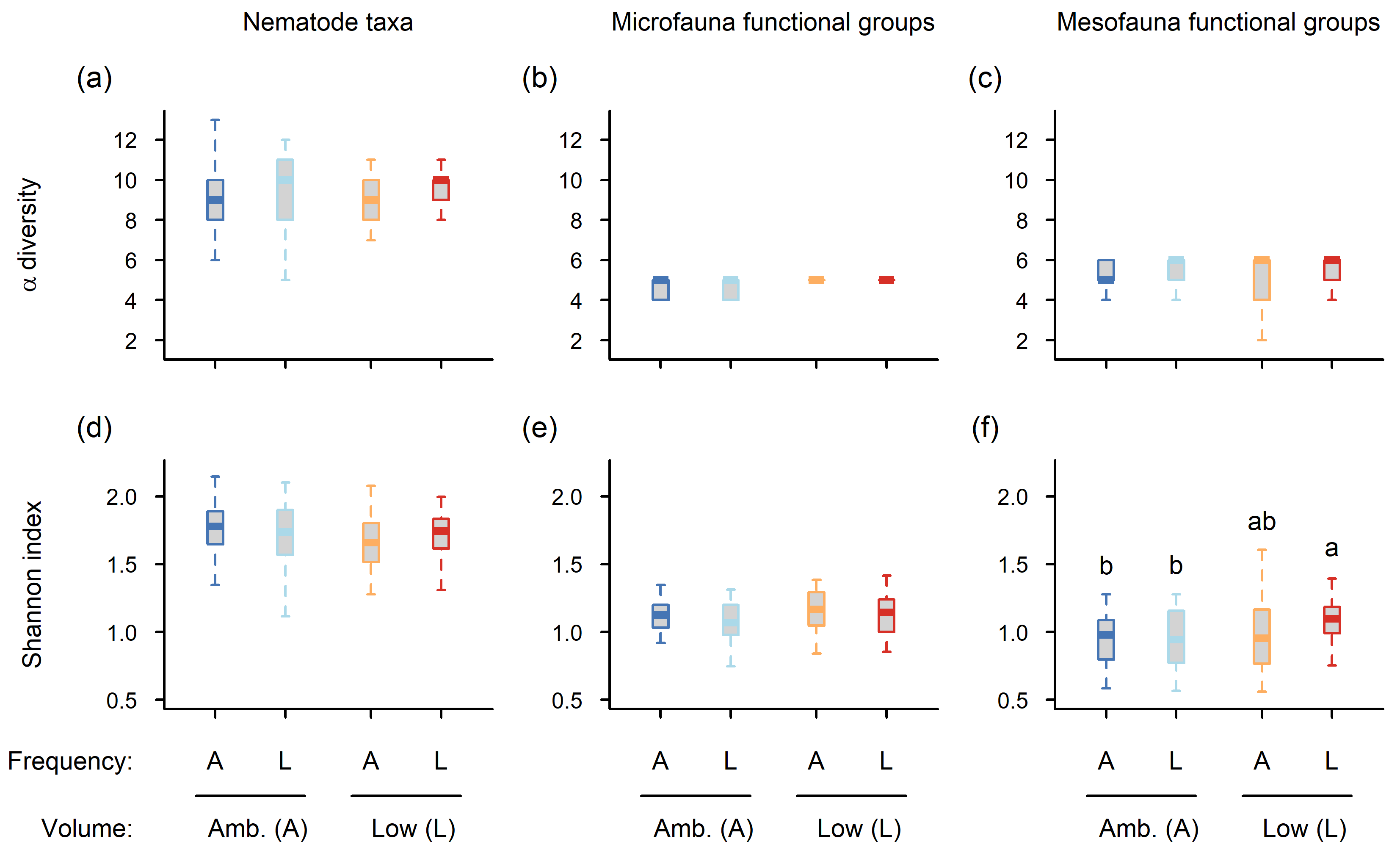


**Figure S1.** Alpha- and Shannon-diversity of nematode taxa, and of functional groups of microfauna and mesofauna, for each of two levels (Ambient (A) and Low (L)) of precipitation volume and frequency. Data were aggregated across island size class, because island size had only minor effects on diversity (Table S4). Boxplots indicate the median (thicker line), the first and third quartiles (lower and upper box boundaries), and the most extreme observations that were up to 1.5 times the interquartile range (hinges). For each volume × frequency combination, N = 30. Within each panel, same letters (or no letters) indicate that differences were not statistically significant (i.e., P > 0.05). Details of the model underpinning the statistical testing are provided in Table S4.

**References**

Bongers T (1990) The maturity index: an ecological measure of environmental disturbance based on nematode species composition. Oecologia 83:14–19.

Clemmensen, K. E. et al. 2013. Roots and associated fungi drive long-term carbon sequestration in boreal forest. Science 339: 1615–1618.

Lagerström, A. et al. 2009. Soil phosphorus and microbial response to a long-term wildfire chronosequence in northern Sweden. Biogeochemistry 95: 199–213.

Wardle, D. A. et al. 1997. The influence of island area on ecosystem properties. - Science 277: 1296–1299.

Wardle, D. A. et al. 2003. Long-term effects of wildfire on ecosystem properties across an island area gradient. Science 300: 972–975.

Wardle, D. A. et al. 2004. Ecological linkages between aboveground and belowground biota. Science 304: 1629.

Wardle, D. A. et al. 2012. Linking vegetation change, carbon sequestration and biodiversity: insights from island ecosystems in a long-term natural experiment. Journal of Ecology 100: 16–30.

Yeates GW, Bongers T, De Goede RG, et al (1993) Feeding habits in soil nematode families and genera-an outline for soil ecologists. Journal of nematology 25:315–331
